# Assessment of T-cell receptor repertoire and clonal expansion in peripheral T-cell lymphoma using RNA-seq data

**DOI:** 10.1038/s41598-017-11310-0

**Published:** 2017-09-12

**Authors:** Qiang Gong, Chao Wang, Weiwei Zhang, Javeed Iqbal, Yang Hu, Timothy C. Greiner, Adam Cornish, Jo-Heon Kim, Raul Rabadan, Francesco Abate, Xin Wang, Giorgio G. Inghirami, Timothy W. McKeithan, Wing C. Chan

**Affiliations:** 1 Department of Pathology, City of Hope National Medical Center, Duarte, 91010 CA United States; 2 Department of Pathology and Microbiology, University of Nebraska Medical Center, Omaha, NE United States; 3 Department of Hematology, Shandong Provincial Hospital affiliated to Shandong University, Jinan, P.R. China; 4 Department of Pathology, Chonnam National University Medical School and Research Institute of Medical Sciences, Gwangju, South Korea; 5 Department of Biomedical Informatics, Columbia University, New York, NY United States; 6 Department of Pathology and Laboratory Medicine, Weill Cornell Medical College, New York, NY United States

## Abstract

T-cell clonality of peripheral T-cell lymphoma (PTCL) is routinely evaluated with a PCR-based method using genomic DNA. However, there are limitations with this approach. The purpose of this study was to determine the utility of RNA-seq for assessing T-cell clonality and T-cell antigen receptor (TCR) repertoire of the neoplastic T-cells in 108 PTCL samples. TCR transcripts, including complementarity-determining region 3 (CDR3) sequences, were assessed. In normal T cells, the CDR3 sequences were extremely diverse, without any clonotype representing more than 2% of the overall TCR population. Dominant clones could be identified in 65 out of 76 PTCL cases (86%) with adequate TCR transcript expression. In monoclonal cases, the dominant clone varied between 11% and 99% of TCRβ transcripts. No unique Vα or Vβ usage was observed. Small T-cell clones were often observed in T- and NK-cell tumors in a percentage higher than observed in reactive conditions. γ chain expression was very low in tumors expressing TCRαβ, but its expression level was high and clonality was detected in a TCRγδ expressing tumor. NK cell lymphoma (NKCL) did not express significant levels of TCR Vβ or Vγ genes. RNA-seq is a useful tool for detecting and characterizing clonal TCR rearrangements in PTCL.

## Introduction

Each mature T-cell expresses a unique T-cell antigen receptor (TCR) which is a combination of either αβ chains or γδ chains. Diversity of the TCR repertoire reflects the initial V(D)J recombination events as shaped by selection by self and foreign antigens. TCRγ rearrangement occurs before the rearrange of the α and β loci, which are associated with αβ/γδ lineage commitment; αβ T cells account for approximately 95% of the T-cell population except for certain extranodal sites such as the epithelium of the gastrointestinal tract.

Next generation sequencing (NGS) is a powerful method for profiling the TCR repertoire, including sequences encoding the complementarity-determining region 3 (CDR3), and provides a broad view of the immune response alterations resulting from perturbations such as infection, vaccination, and cancer^1, 2^. Several different NGS platforms, using genomic DNA or cDNA from T cells, have been used for sequencing the TCR. Whole transcripome sequencing (RNA-seq) provides adequate coverage to study the TCR repertoire and has been utilized to profile tissue-resident T cell repertoires^3^.

Peripheral T-cell lymphomas (PTCL) constitute about 10% of non-Hodgkin lymphomas and comprise a heterogeneous group of relatively rare and aggressive malignancies derived from mature T cells^4^. PTCL is characterized by clonal expansion of T cells and can be further classified into many subtypes based on their distinct morphological, immunophenotypic, molecular, and clinical differences, including angioimmunoblastic T-cell lymphoma (AITL), anaplastic large cell lymphoma (ALCL), and adult T-cell leukemia/lymphoma (ATLL). Pathological diagnosis remains challenging, and there is a large of group of cases that cannot be further classified and are grouped under PTCL, not otherwise specified (PTCL-NOS). TCR rearrangement assays improve clinical diagnostics by demonstrating the presence of dominant clones in PTCL, and the unique rearrangement is useful in monitoring minimal residual disease^5–7^. Furthermore, preferential TCR usage in a T-cell lymphoma subtype may suggest immune perturbations and antigen selection that predispose to lymphomagenesis^8^. Although TCRαβ-expressing tumors represent 90% of T-cell malignancies, T-cell clonality is routinely evaluated with a PCR-based method to detect TCRγ and, less frequently, β chain rearrangement using genomic DNA.

The TCRα and β loci are too complex and cumbersome for routine DNA clonal analyses, but flow cytometry can also detect TCRβ gene usage and thus imply clonality^9, 10^. However, these approaches are also limited by the lack of sequence information. RNA-seq provides information about the usage of variable (V), diversity (D) if applicable, and joining (J) regions of the TCRα, β, γ, and δ chains and yields the unique sequence of the CDR3, which includes the V(D)J junctions as well as N-nucleotides added by terminal deoxynucleotidyl transferase (TdT). We report here the utility of RNA-seq in assessing T-cell clonality and in analyzing the TCR repertoire in different PTCL subtypes.

## Materials and Methods

### Patient specimen and data source

We analyzed RNA-seq data from our laboratory and other sources (Supplementary Table S1), including 40 angioimmunoblastic T-cell lymphomas (AITL), 35 anaplastic large cell lymphomas (ALCL), 17 PTCL, not otherwise specified (PTCL-NOS), 15 NK cell lymphomas (NKCL), 1 γδ-T cell lymphoma (γδ-TCL), and 6 ALCL cell lines^11–15^. Informed consent was obtained from all patients for the RNA-seq experiments. Data from normal T cells were obtained from publicly available resources^16^. All experiments in this study were performed in accordance with the relevant guidelines and regulations, and were approved by the Institutional Review Boards of the University of Nebraska Medical Center (#543–09-ep) and City of Hope Medical Center (#13478).

### Whole transcriptome sequencing

Whole transcriptome sequencing was performed as previously described^11, 14^.

### Identification of CDR3 sequences

MiXCR (v1.2)^17^ was used to extract apparent CDR3 sequences from RNA-seq data, but the program erroneously extracted many non-TCR reads. For example, somatically mutated immunoglobulin κ CDR3 regions may resemble those of TCRα. Therefore, we performed additional filtering steps to exclude sequences transcribed from non-TCR loci. First, reads that include apparent CDR3 sequences were aligned against the human reference genome (hg38) using BLAT (version 34, default settings)^18^. Reads that had a >80% match with non-TCR or different TCR regions and that did not alternatively match with the MiXCR-reported TCR regions according to the BLAT results were removed. Second, the CDR3 nucleotide sequence of each clone was also aligned against the human reference genome (hg38) using BLAT, and clone sequences with nearly complete matches (>90% match) were removed because CDR3 sequences are expected to consist of sequences from V and J genes and several random bases in between. Third, in order to avoid noise from unknown sequences, clones with CDR3 sequences that were recurrently found in more than eight patient cases were removed. The script that performs the described filtering process is available at https://github.com/littlegq/MixcrFilter. The frequencies of the 10 most frequent CDR3 were determined in each case as illustrated.

### Quantification of TCR transcripts

RNA-seq reads were mapped to the human reference genome (hg38) with TopHat 2 (v2.0.10)^19^. Fragments per kilobase of transcript per million mapped reads (FPKM values) were calculated using the “cuffnorm” from Cufflinks (v2.2.1)^20^ program with default parameters. The TCR gene annotations were obtained from the Comprehensive gene annotation from the GENCODE database^21^. The number of reads containing CDR3 sequences per million mapped reads (RPM) was used to assess the overall TCRα and TCRβ chain transcript levels:

1 $$RP{M}_{\alpha /\beta }=\frac{{\sum }_{i=1}^{n}{C}_{i}}{N}\times {10}^{6}\,,$$

where ${C}_{i}$ is the number of reads containing each TCRα or TCRβ gene as determined by MiXCR, $N$ is the number of total RNA-seq reads that were mapped to the human genome by TopHat 2 or a similar RNA-seq aligner.

### Assessment of clonality

We assess clonality by the absolute proportion of a clone or the relative ratio of the two largest clones to the third largest one. For non-clonal tissues, the percentage of T-cells sharing the same CDR3 sequences is not expected to exceed 2% according our data. However, with limited read coverage, the observed fraction diverges from the true fraction of transcripts. For a unique CDR3 covered by $n$ reads, the standard error $SE\,\,$of the observed clone proportion $p$ can be calculated as:

2 $$SE=\sqrt{\frac{p(1-p)}{n}},$$

In practice, we observed 500–2400 CDR3-containing reads (median value: 1484) in the majority of samples. This number was used to estimate the error (Supplementary Figure S1A) around a range of possible thresholds, as measured by the ratios of SE and the corresponding threshold of minimal fraction. To balance a low error rate and high sensitivity, we set the threshold for the minimum size of the dominant clones (top 1 + 2) to be 10%.

For samples with very low tumor content, which is common for AITLs, clonality could also be determined by the ratio of the top 2 clones to the 3^rd^ largest one. In 89.4% of monoclonal samples as determined by the “10% rule”, this ratio was larger than 10, which was used as a cutoff for the dominant clones (Supplementary Figure S1B).

In summary, a case was considered to have a clonal population if the sum of transcripts from the top two CDR3α or CDR3β clones was at least 10 times larger than the third largest or >10% of all observed TCRα or β transcripts. In addition, a monoclonal case was considered biallelic if the transcripts from the second largest CDR3α or CDR3β clone were at least 5 times larger than the third largest.

We also explored alternative criteria based only on the normalized gene transcript levels (measured as FPKM in this study) as calculated by the RNA-seq analytic pipeline. Similar to the CDR3-based criteria, a case was considered monoclonal if the sum of FPKM of the top two Vα or Vβ genes was at least 10 times larger than the third highest. If there was a conflict between clonality assessment based on CDR3 versus V-usage data, the CDR3 assessment was adopted.

Our definition of the threshold is empirical, and more extensive experience is needed to identify the best threshold for diagnosis.

### Mutation analysis

RNA sequencing reads were mapped against the human reference genome (hg19) using Tophat (v2.0.10)^19^ with default settings. VarScan (v2.3.6)^22^ was used to call the variants. Each variant was required to be covered by at least 10 reads, including at least 4 variant-supporting reads, and with a minimal variant frequency of 5%. SNPs in dbSNP (v138) database, except those with minor allele frequency <1% (or unknown), were excluded.

### Identification of EBV infection in PTCL using RNA-seq

Sequences were mapped against the EBV genome (AJ507799.2) using Burrows-Wheeler Aligner (v0.7.5)^23^ and analyzed with SAMtools (v0.6.1)^24^. Samples with 100 or more EBV-derived reads were classified as EBV-positive (Supplementary Figure S2).

## Results

**Figure 1:**
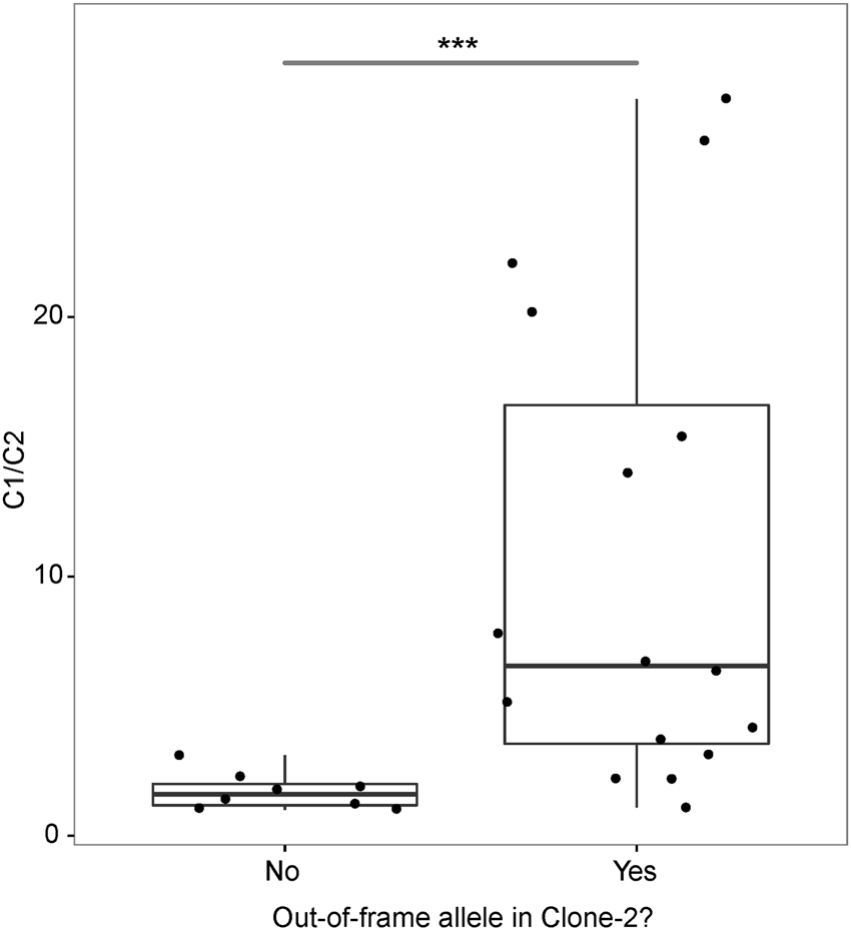
Ratio of CDR3 transcript levels of the first and second largest clones (C1/C2) in monoclonal PTCL cases with two TCRα or TCRβ clones.

We utilized two analytical strategies to detect the clonotypes from RNA-seq data. One was to directly quantify the transcripts in FPKM values to show usage of individual V genes. The other method was to identify the clonotypes based on the sequence of the CDR3, which contains unique V-N-(D)-N-J junctions, nontemplated nucleotide insertions, and base deletions. These two strategies provided generally concordant assessment of monoclonality (Supplementary Table S2). In a few cases, however, clonality of TCRα and TCRβ was discrepant. 21 cases showed two clonal transcripts for either Vα or Vβ, and two thirds of them contain one nonproductive allele (containing a frameshift or nonsense codon) (Supplementary Table S3), suggesting the possibility of dual receptor expression^25, 26^ in some of the cases, resulting from transcription from biallelic in-frame rearrangements. Interestingly, among the biallelic clonal TCRα or TCRβ transcripts, the ratio between the top 2 clones (C1/C2) is significantly higher when the second-largest clone contains nonproductive CDR3 sequences (p = 0.0008, Welch two sample t-test; Figure 1), which may be due to the nonsense-mediated mRNA decay mechanism^27^.

Vα and Vβ transcripts were abundant in AITL and PTCL-NOS cases compared to ALCL and NKCL cases. Although Vγ and Vδ transcripts were rare in these entities, a single γδ-TCL case showed low expression of TCR-Vα and -Vβ but clonal TCR-Vγ and -Vδ expression; this was confirmed by PCR analysis of TCRγ chain (Supplementary Figure S3).

**Figure 2:**
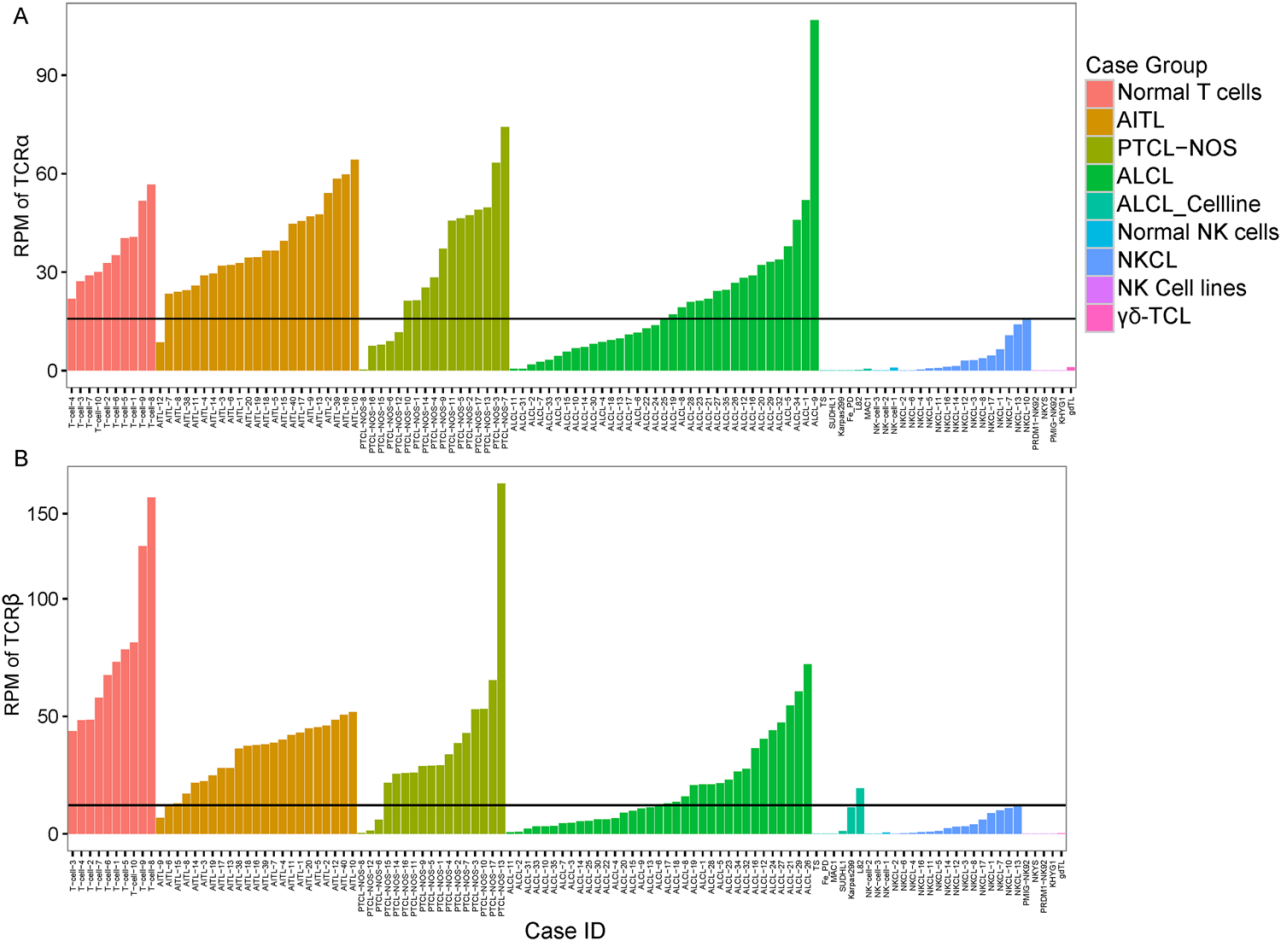
Reads containing CDR3 sequences per million mapped reads (RPM) of each sample. The black bars represent the cutoffs of TCRα (**A**) and TCRβ (**B**) transcripts in tumors.

Unlike normal tonsillar T-cell populations, multiple small CDR3 clones in addition to the presumed neoplastic ones were often observed in tumors and were particularly prominent if a limited number of normal infiltrating T-cells were present. When the neoplastic TCR was not expressed or few tumor cells were present, such clones could appear as the largest clones accounting for a large fraction of the total and be falsely considered as the neoplastic clone. To avoid such a situation, we tried to derive a threshold for TCR transcript levels below which the assessment was considered unreliable. There was low expression of Vα and Vβ in NKCL and γδ-TCL, likely from infiltrating non-neoplastic T cells. Thus, we used NKCL and γδ-TCL samples to set the threshold for selecting cases with sufficient TCRα and TCRβ expression for subsequent analysis. Cases with lower expression of TCRα and TCRβ than NKCL (RPM: TRA < 15.82 or TRB < 12.21) were excluded (three PTCL-NOS, five ALCL cell lines, and 13 ALCL cases; Figure 2).

**Figure 3:**
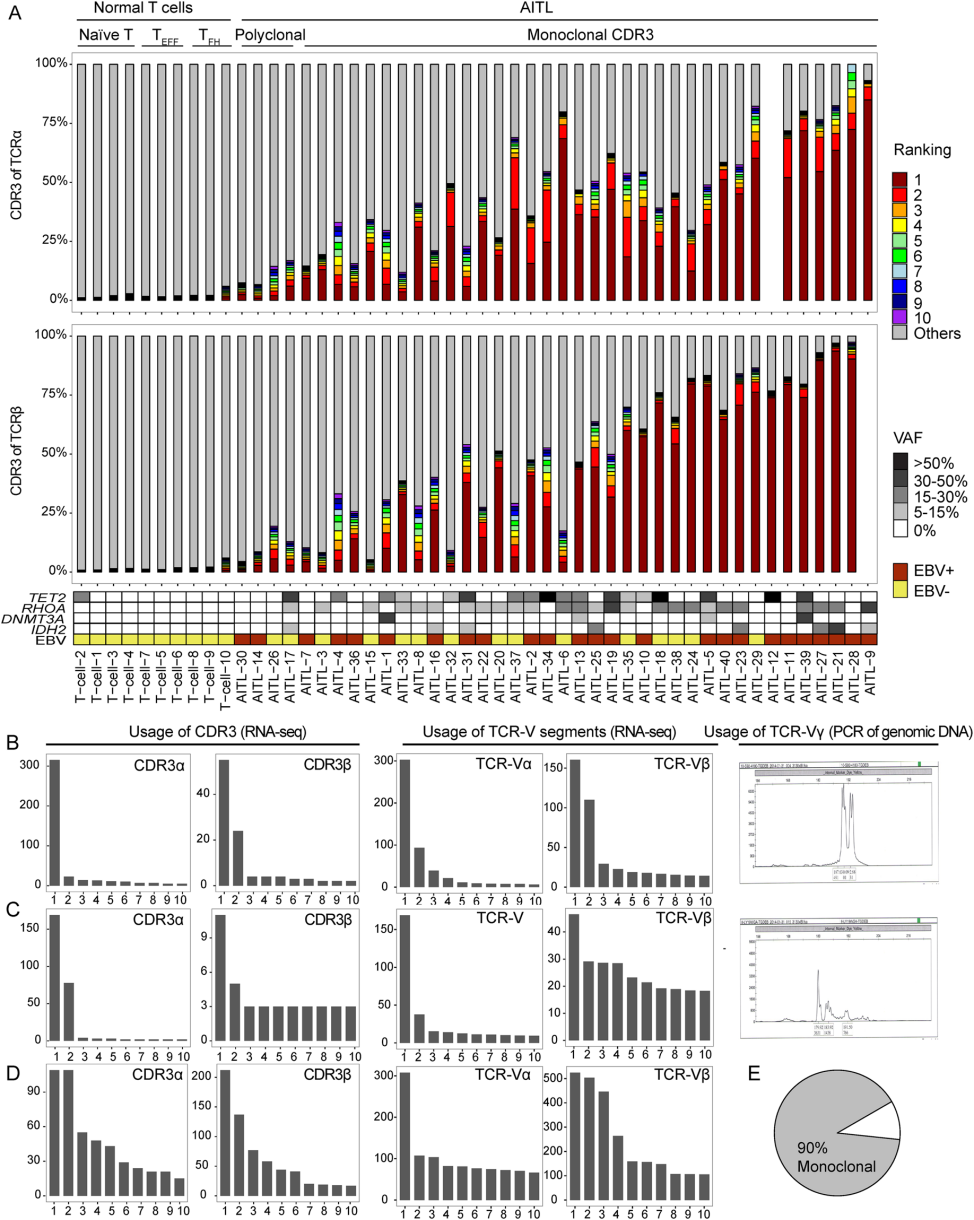
Clonal configuration of TCR transcripts in AITL. (**A**) Contribution of CDR3α and CDR3β sequences to the TCR repertoire in 10 normal T cell sets and 40 AITL samples. Each bar represents an individual clonotype, with red to violet showing the first to tenth ranked predominant clonotypes. Grey represents the rest of the identified clonotypes in the sample. Data are not shown for TCR chains with transcript levels below the threshold. Examples of monoclonal cases, AITL-22 (panel **B**) and AITL-32 (panel **C**), and a polyclonal case, AITL-1 (panel **D**) with ranked CDR3-containing reads (counts), V gene usage (FPKM), and spectrum of rearranged γ chains by PCR. (**E**) Frequency of monoclonal cases of AITL.

**Figure 4:**
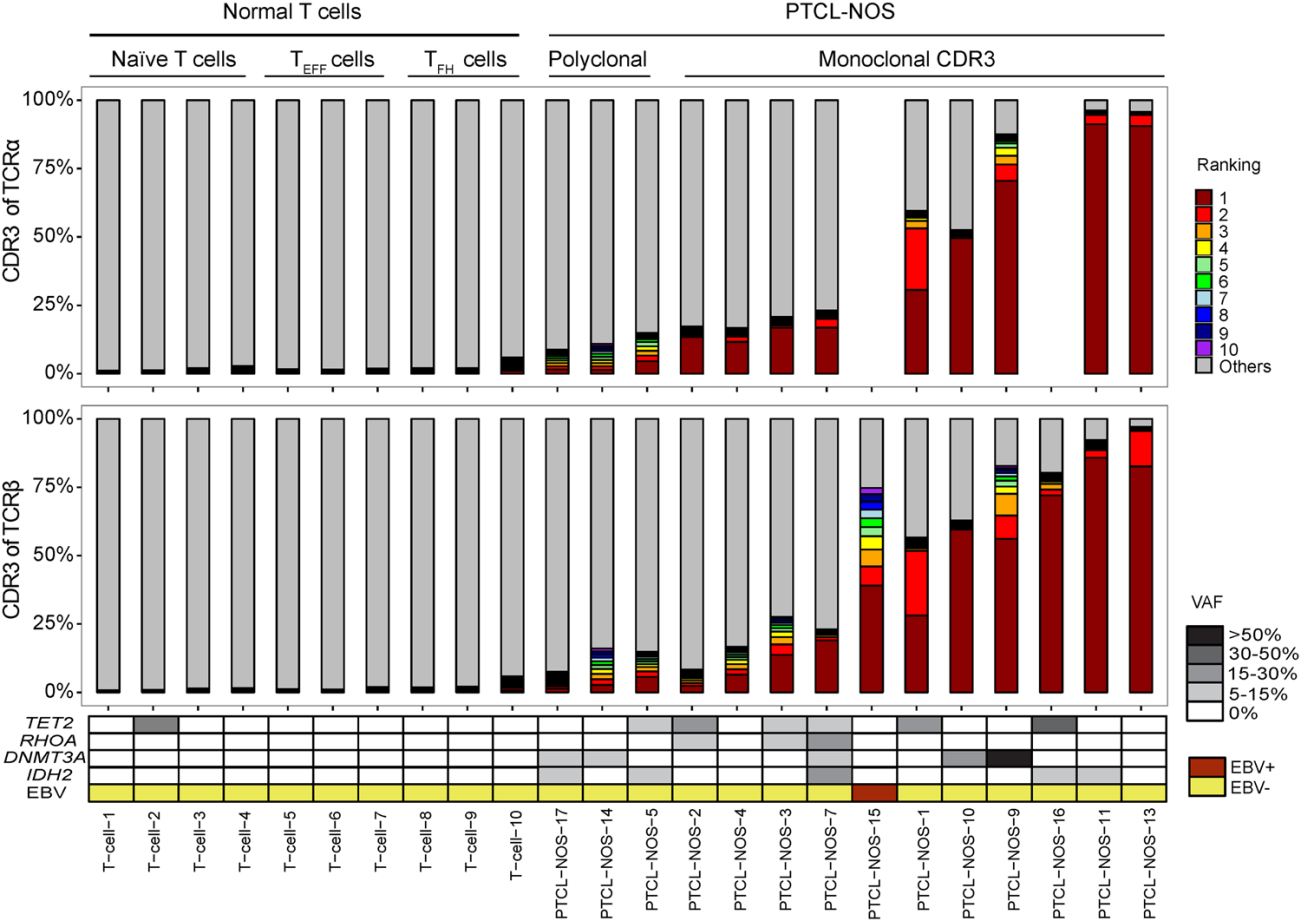
Clonal configuration of TCR transcripts in PTCL-NOS. See the legend of Figure 3 for details.

Dominant clones with unique CDR3 sequences were identified in most AITL (36/40) (Figure 3) and PTCL-NOS (11/14) cases (Figure 4). The abundance of the clonal transcript varied in monoclonal cases (between 11.5% and 95.6% of TCRβ CDR3 transcripts), probably due to the variable fraction of tumor cells and variable TCR transcript levels in neoplastic and non-neoplastic T cells. We have data on the frequent mutations (*IDH2*, *TET2*, *DNMT3A*, and *RHOA)* in AITL^28–31^. Most monoclonal AITL cases (31/36) harbored at least one such mutation, whereas most polyclonal cases (3/4) were negative (Figure 3), which suggests a significant association between TCR clonality and AITL-associated mutations (p = 0.02, Fisher’s exact test). Failure to detect a clonal transcript could be due to loss of TCR transcription or to low tumor content. Notably, 5 AITL cases showed a dominant clone based on TCRα but not TCRβ transcripts (Figure 3). This suggests that the frequent loss of TCR and surface CD3 expression in AITL^32^ can often be explained by loss of TCR gene expression in the malignant clone.

**Figure 5:**
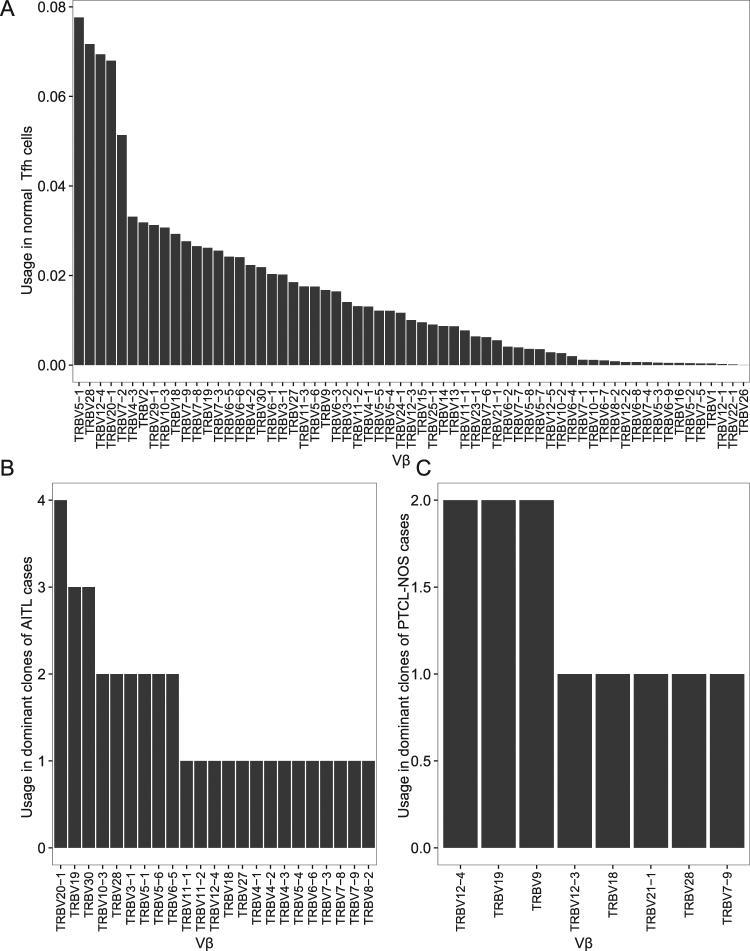
Usage of TCR-Vβ in normal T_FH_ cells (**A**), dominant AITL clones (**B**), and dominant PTCL-NOS clones (**C**).

We also compared the Vβ gene usage in malignant clones with the Vβ usage in several normal tonsillar T-cell populations. Preferential usage of particular Vα and Vβ is similar among naïve T cells, T_FH_ cells, and T_EFF_ cells. In tumor samples, we only consider the clonal V genes being used by tumor cells. Vβ genes often used by the tumor clones were also frequently used by normal T-cells (Figure 5 and Supplementary Figure S4). For example, *TRBV20–1*, one of the most commonly used genes, was also frequently used in the dominant clones in AITL. *TRBV9*, *TRBV12–4*, and *TRBV19*, each of which was found in the dominant clones of two PTCL-NOS cases, are also commonly used in normal T-cells. Thus, we did not detect distinct preferential usage of a Vβ gene in AITL or PTCL-NOS. AITL frequently harbors Epstein-Barr virus (EBV) infection of B cells in the microenvironment. EBV-derived transcripts could be readily detected in many AITLs (24/40). Although CD8^+^ T cells responding to dominant EBV epitopes preferentially express certain TCR-Vβ genes^33, 34^, we did not observe a high frequency of these Vβ transcripts in infected cases.

**Figure 6:**
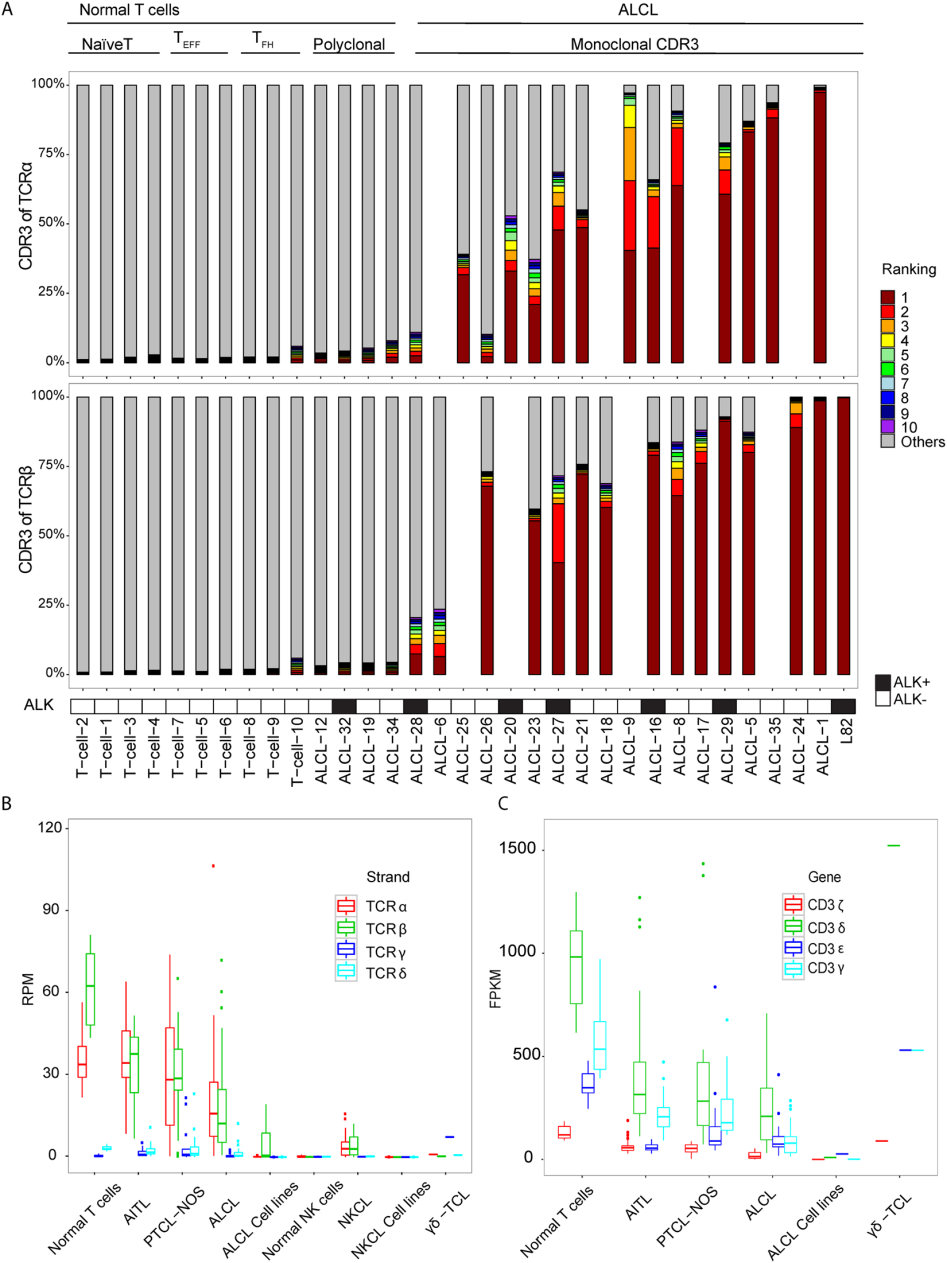
Some ALCL cases showed clonal TCR expression at the mRNA level. (**A**) Contribution of CDR3α and CDR3β sequences to the TCR repertoire in 10 normal T cell sets, 22 ALCL samples, and one ALCL cell line. (**B**) Expression of α, β, γ, and δ chains of TCR in primary cells, cell lines, and different subtypes of PTCL samples. (**C**) Expression of γ, δ, ε, and ζ chains of CD3 in primary cells, cell lines, and different subtypes of PTCL samples. Samples that do not express TCR transcripts (below the cut-off according to Figure 2) were excluded.

ALCL comprises a heterogeneous group of CD30^+^ PTCLs with systemic or primary cutaneous presentation. ALCLs lack surface expression of a TCR and the TCR-associated CD3 complex^35^. Little is known regarding the expression of TCR mRNAs in ALCL. Expression of Vα or Vβ transcripts varied among ALCL samples and cell lines but was generally low (Figure 6B), and 13 cases were excluded because of low transcript level. We could detect clonal CDR3 transcripts in 18 of 22 ALCL cases with transcript levels above threshold (Figure 6A). To confirm that ALCL tumor cells can indeed express Vα or Vβ transcripts, we examined the expression of Vα and Vβ in ALCL cell lines. TCR mRNA was only detected in L82, an ALK-positive line that showed only clonal Vβ CDR3 expression. 9/18 cases also showed expression of only CDR3α or CDR3β. Thus, TCR transcription was frequently abnormal in ALCL but cannot fully explain the lack of cell-surface expression of the TCR.

## Discussion

The clonal rearrangement of TCR genes is useful in supporting the diagnosis of a T-cell lymphoma. The current clinical approach consists of PCR amplification and fragment analysis of the VJ junction region (CDR3) of the rearranged *TCRG* and, sometimes, the VDJ region of the *TCRB* locus. Analysis of the size of these amplified regions in normal polyclonal T-lymphocytes reveals numerous peaks with a Gaussian distribution of their lengths, whereas in tumors, the PCR products are present as a single major peak (monoallelic) or as two peaks (biallelic arrangement). T-cells follow a pattern of T-cell receptor rearrangement, with *TCRD* rearranged first, followed by *TCRG* and incomplete rearrangement of *TCRB* (Dβ-Jβ)^36, 37^. All mature T-cells have *TCRG* rearrangement; thus, it is a good clonal marker for all T-cell lymphomas derived from mature T-cells. However, *TCRG* transcripts are present only in γδ T-cells. In AITL and PTCL-NOS, clonal rearrangements of TCR genes are reported to be detected in about 80% of cases^38^.

In the current study, we evaluated the clonal expansion and TCR repertoire at the nucleotide level in 108 PTCLs (40 AITLs, 35 ALCLs, 17 PTCL-NOS, 15 NKCLs, and one γδ-TCL). V gene transcripts and CDR3 sequences were extracted from RNA-seq data to assess clonality. The assessment results that were based on V gene usage and CDR3 sequences were mostly concordant (Supplementary Table S2). However, there were still a few cases which were assessed to be monoclonal based on CDR3 sequences but polyclonal based on V-usage. This could result from reads that were falsely or ambiguously mapped to parts of V genes. For CDR3 sequences, we filtered out these reads based on global alignment results using BLAT, but they might still be counted in the V-usage results. Therefore, the CDR3 sequences should serve as a more reliable basis for clonality assessment, whereas the V-usage method could be used for a quick assessment based on a normalized table of gene transcript levels. Both methods were based on general RNA-seq technology, with limited read coverage on the TCR genes. Thus, only major clones (>0.1%) were expected to be detected in samples with active TCR transcription. Therefore, RNA-seq data could be used for clonality analysis for tumor samples, but for other purposes which require more complete TCR repertoire profiles, techniques enabling target deep sequencing should be considered^1–3^.

Monoclonal CDR3 sequences were found in 66 PTCL samples, accounting for 86% out of a total of 77 samples with sufficient TCR expression. This result agrees with a recent study, which explored the TCR clonality of AITLs and PTCL-NOS, and found that 91% were clonal^39^. The slightly higher proportion of clonal samples may be because tumor cells were enriched by antibodies before sequencing.

At least one clonal transcript was observed in most of the cases of PTCL with sufficient expression of TCR transcript levels. We have not observed any preferential Vβ usage in the tumors examined, which would suggest the role of antigen-driven lymphomagenesis. Interestingly, in a few cases, there was in-frame expression of two clonal TCRα and TCRβ transcripts suggesting that two TCR molecules could be expressed by the tumor cells as observed in some cases of PTCL.

While concordant TCR-Vα and TCR-Vβ clonal expression was often observed in AITL and PTCL-NOS, one of these was missing in some of the cases. This is particularly prominent in ALCL with 9 of 18 cases showing expression of only CDR3α or CDR3β. It is unclear why surface TCR expression is absent in other ALCL cases with TCR mRNA expression. For surface expression, TCR heterodimers must be noncovalently bound to all CD3 subunits^40, 41^. In particular, lack of CD3γ severely reduces TCR surface expression^40^. The very low expression of CD3 subunits and in particular CD3γ in ALCL (Figure 6C) potentially may explain the abnormal TCR expression. The mechanisms behind the abnormalities in TCR expression require further investigation. Absent or decreased surface TCR and CD3 protein expression was found in the majority of AITL cases^32^. Our data suggest that in some cases, this may result from loss of expression of one or both of the α and β TCR subunits; in other cases, it may be due to low expression of CD3 subunits (Figure 6C), as postulated for ALCL. The frequent partial or complete loss of surface TCR expression in AITL is surprising, given the importance of TCR signaling in T-cell survival. Activating mutations affecting genes involved in TCR signaling are frequent in AITL^11, 42^. These activating mutations presumably allow signaling for survival and proliferation in the absence of tonic TCR signaling.

BCR and TCR rearrangement can also be investigated by NGS using genomic DNA. This is generally performed after PCR amplification of the CDR3 of the locus of interest^43, 44^. However, the massive number of PCR reactions that are needed and the difficulty of ensuring unbiased amplification make such an analysis difficult to replicate in academic molecular diagnostic laboratories. RNA-seq is far easier to perform but will miss rearrangements in TCR loci that are not expressed. However, this is a very useful approach in determining the TCRα and TCRβ repertoire in non-neoplastic infiltrates in the tumor microenvironment or in inflammatory settings.

We have demonstrated that RNA-seq is a useful approach in determining clonality in PTCL. Some cases do not show clonal transcript expression due to either low tumor content or abnormal biology, which is much more frequent in ALCL. The sequencing approach allows the study of the TCR repertoire of the tumor cells and infiltrating normal T-cells and potentially, the monitoring of minimal residual disease.

### Data availability

The RNA-seq data used in this study can be accessed via the accession numbers given by the original publications as listed in Supplementary Table S1, or via Sequence Read Archive (https://www.ncbi.nlm.nih.gov/sra) with accession number SRP099016.

## Electronic supplementary material

Supplementary Information
