# Supplementary material for: Assessment of T-cell receptor repertoire and clonal expansion in peripheral T-cell lymphoma using RNA-seq data
Source: Sci Rep. 2017 Sep 12;7:11301. doi: 10.1038/s41598-017-11310-0 (PMC5595876; doi:10.1038/s41598-017-11310-0)
Supplement: Supplementary file 1 — Supplementary Information [file 41598_2017_11310_MOESM1_ESM.pdf]

Assessment of T-cell receptor repertoire and clonal expansion in peripheral T-cell lymphoma using RNA-seq data

Qiang Gong[1,7], Chao Wang[1,2,3,7], Weiwei Zhang[2,7], Javeed Iqbal[2], Yang Hu[2], Timothy C. Greiner[2], Adam Cornish[2], Jo-Heon Kim[1,4], Raul Rabadan[5], Francesco Abate[5], Xin Wang[3], Giorgio G. Inghirami[6], Timothy W. McKeithan[1], Wing C. Chan[1*]

[1]Department of Pathology, City of Hope National Medical Center, Duarte, CA; [2]Department of Pathology and Microbiology, University of Nebraska Medical Center, Omaha, NE; [3]Department of Hematology, Shandong Provincial Hospital affiliated to Shandong University, Jinan, P.R. China; [4]Department of Pathology, Chonnam National University Medical School and Research Institute of Medical Sciences, Gwangju, South Korea; [5]Department of Biomedical Informatics, Columbia University, New York, NY; [6]Department of Pathology and Laboratory Medicine, Weill Cornell Medical College, New York, NY; [7]These authors contributed equally.

List of supplementary figures and tables:

- Figure S1 Cutoffs for clonal assessement.
- Figure S2 Number of reads from EBV genomes in each sample.
- Figure S3 TCR-Vγ transcription levels in a γδ-T cell lymphoma case.
- Figure S4 Usage of TCR-Vβ in normal effector T and native T cells.
- Table S1 List of samples used in this study.
- Table S2 Clonal assessment based on TCR CDR3 sequences and variable segment
- usage.
- Table S3 List of samples with biallelic TCR strands.

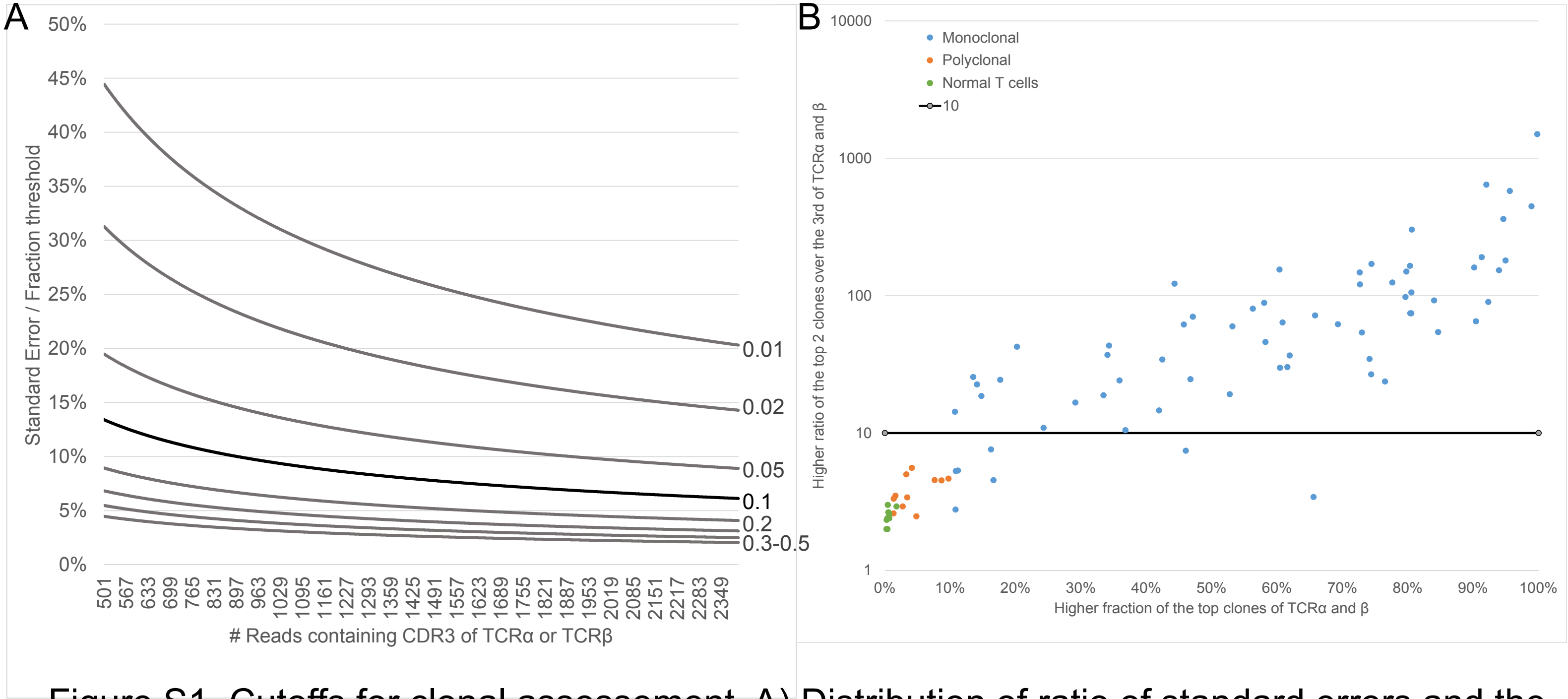

Figure S1. Cutoffs for clonal assessement. A) Distribution of ratio of standard errors and the percentage cutoff above which a clone was considered clonal. B) Distribution of fraction of the largest two clones and their ratio over the third largest clones.

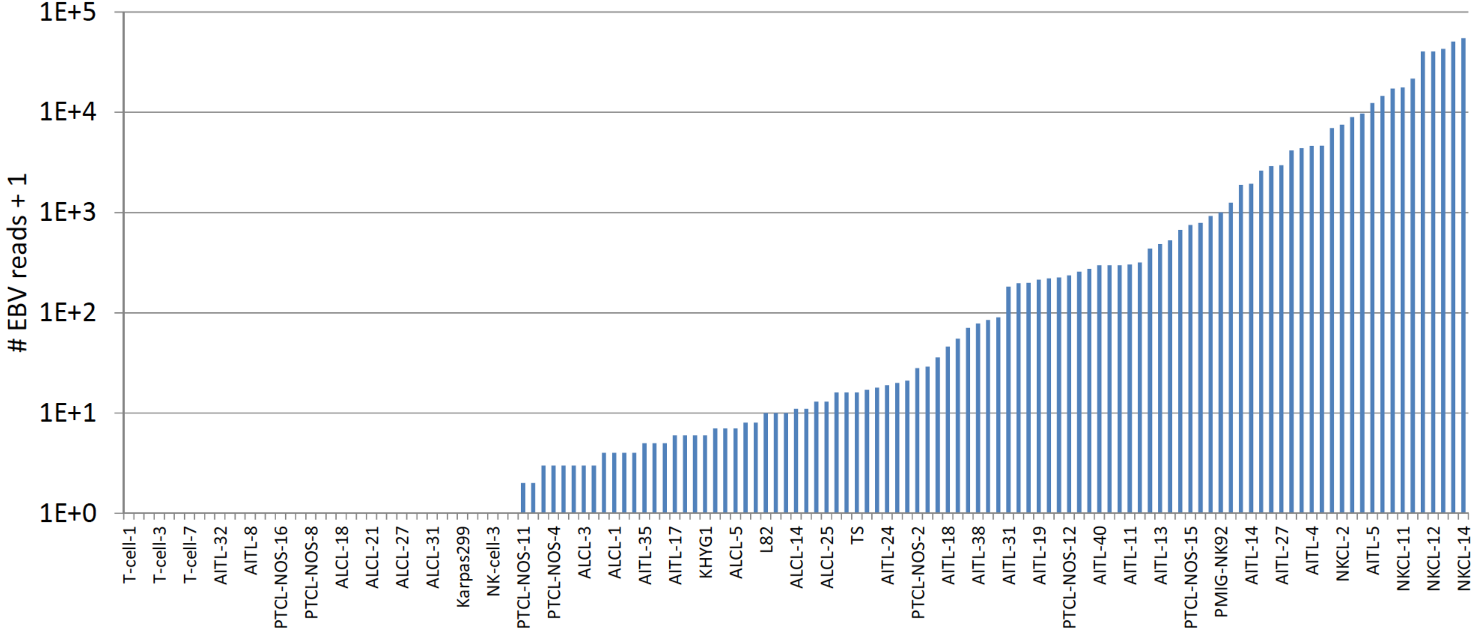

Figure S2 Number of reads from EBV genomes in each sample.

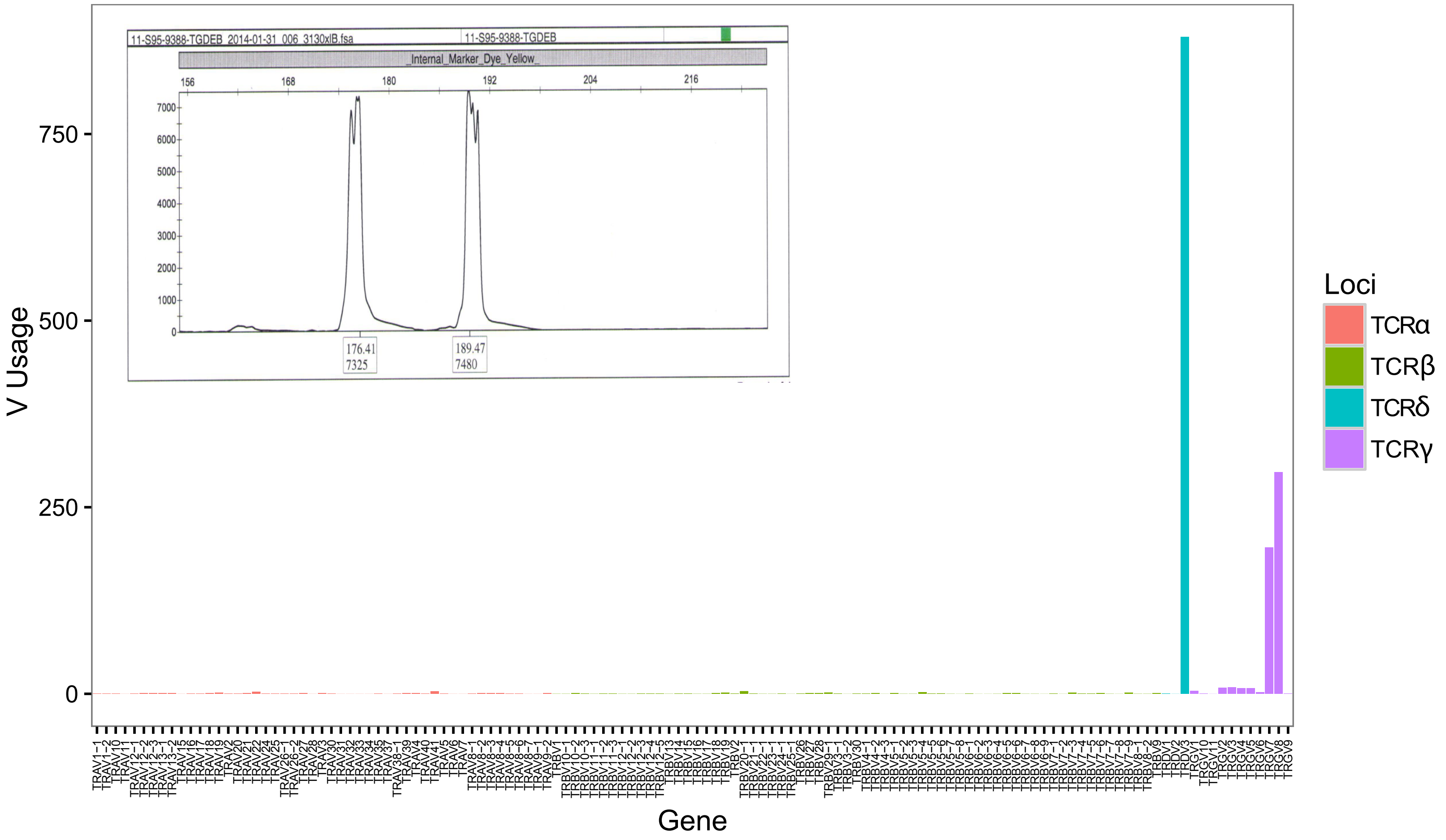

Figure S3 TCR-Vγ transcription levels in a γδ-T cell lymphoma case. The barplot shows usage of TCR-Vα, TCR-Vβ, TCR-Vγ, and TCR-Vδ as determined on RNA-seq data. The inner panel shows the peaks of the PCR results of the TCRγ chain.

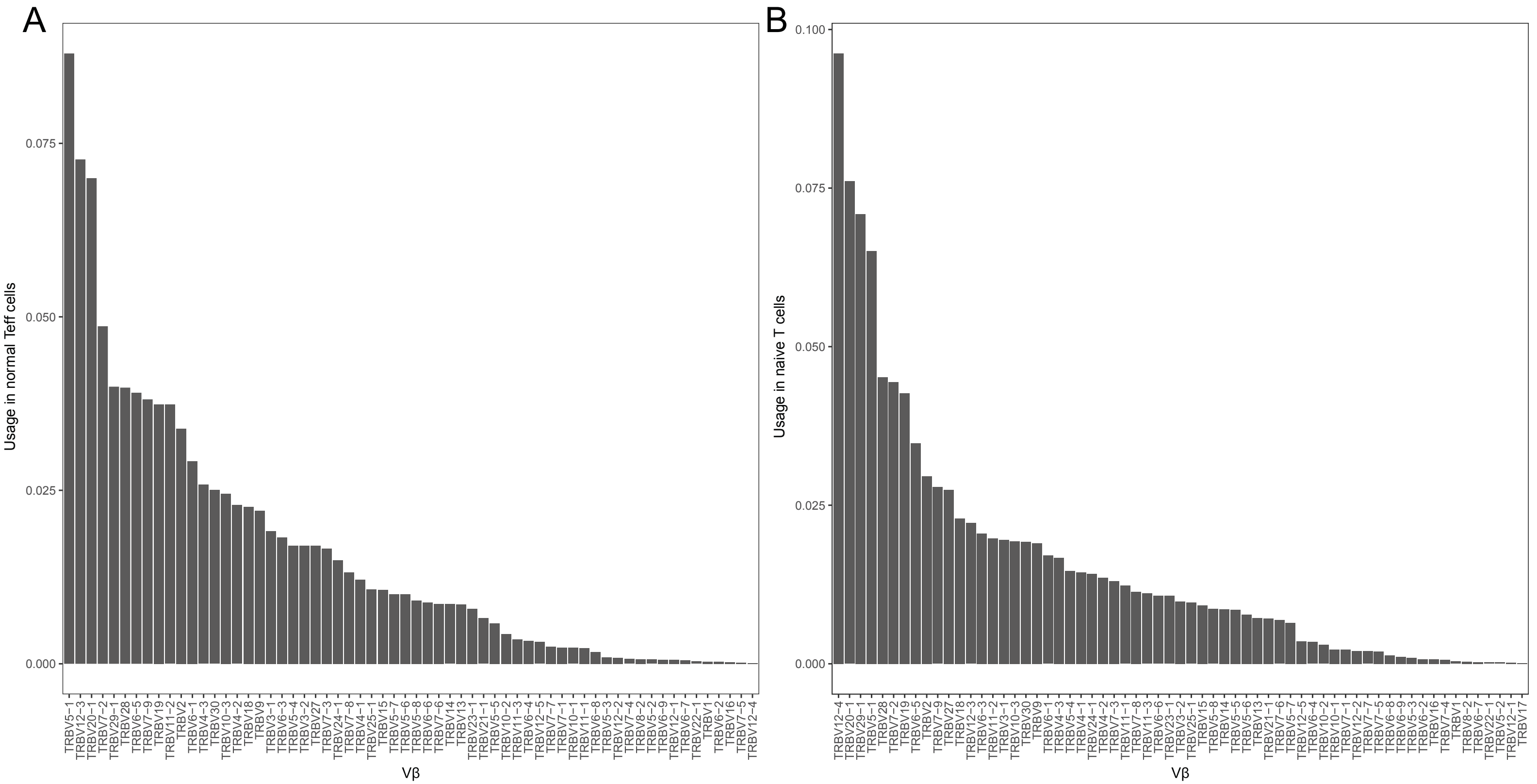

Figure S4 Usage of TCR-Vβ in normal effector T (A) and naive T cells (B).

Table S1 List of samples used in this study.

Sample ID	Subtype	Read Count (Millions)	Source
AITL-1	AITL	57.6	Palomero et al, 2014 (Ref. 14)
AITL-2	AITL	69.5	Palomero et al, 2014 (Ref. 14)
AITL-3	AITL	62.1	Palomero et al, 2014 (Ref. 14)
AITL-4	AITL	68.2	Palomero et al, 2014 (Ref. 14)
AITL-5	AITL	62.8	Palomero et al, 2014 (Ref. 14)
AITL-6	AITL	66.4	Palomero et al, 2014 (Ref. 14)
AITL-7	AITL	74.7	Palomero et al, 2014 (Ref. 14)
AITL-8	AITL	67.6	Palomero et al, 2014 (Ref. 14)
AITL-9	AITL	114.1	Palomero et al, 2014 (Ref. 14)
AITL-10	AITL	62.3	Palomero et al, 2014 (Ref. 14)
AITL-11	AITL	68.4	Palomero et al, 2014 (Ref. 14)
AITL-12	AITL	76.2	Yoo et al, 2014 (Ref. 13)
AITL-13	AITL	67.6	Yoo et al, 2014 (Ref. 13)
AITL-14	AITL	93.6	Yoo et al, 2014 (Ref. 13)
AITL-15	AITL	96.9	Yoo et al, 2014 (Ref. 13)
AITL-16	AITL	91.7	Yoo et al, 2014 (Ref. 13)
AITL-17	AITL	59.9	Yoo et al, 2014 (Ref. 13)
AITL-18	AITL	72.6	Yoo et al, 2014 (Ref. 13)
AITL-19	AITL	61.2	Yoo et al, 2014 (Ref. 13)
AITL-20	AITL	54.8	Yoo et al, 2014 (Ref. 13)
AITL-21	AITL	115.7	Rohr et al, 2015 (Ref. 11)
AITL-22	AITL	117.8	Rohr et al, 2015 (Ref. 11)
AITL-23	AITL	102.7	Rohr et al, 2015 (Ref. 11)
AITL-24	AITL	126.2	Rohr et al, 2015 (Ref. 11)
AITL-25	AITL	110.5	Rohr et al, 2015 (Ref. 11)
AITL-26	AITL	109.3	Rohr et al, 2015 (Ref. 11)
AITL-27	AITL	108.8	Rohr et al, 2015 (Ref. 11)
AITL-28	AITL	84.8	Rohr et al, 2015 (Ref. 11)
AITL-29	AITL	101.6	Rohr et al, 2015 (Ref. 11)
AITL-30	AITL	98.4	Rohr et al, 2015 (Ref. 11)
AITL-31	AITL	108.4	Rohr et al, 2015 (Ref. 11)
AITL-32	AITL	128.2	Rohr et al, 2015 (Ref. 11)
AITL-33	AITL	128.3	Rohr et al, 2015 (Ref. 11)
AITL-34	AITL	110.0	Rohr et al, 2015 (Ref. 11)
AITL-35	AITL	128.4	Rohr et al, 2015 (Ref. 11)
AITL-36	AITL	103.3	Rohr et al, 2015 (Ref. 11)
AITL-37	AITL	95.8	Rohr et al, 2015 (Ref. 11)
AITL-38	AITL	79.4	Rohr et al, 2015 (Ref. 11)
AITL-39	AITL	95.8	Rohr et al, 2015 (Ref. 11)
AITL-40	AITL	87.7	Rohr et al, 2015 (Ref. 11)
gdTL	γδ-TCL	92.9	New data

ALCL-1	ALCL	43.0	Crescenzo et al, 2015 (Ref. 15)
ALCL-2	ALCL	47.2	Crescenzo et al, 2015 (Ref. 15)
ALCL-3	ALCL	38.6	Crescenzo et al, 2015 (Ref. 15)
ALCL-4	ALCL	54.4	Crescenzo et al, 2015 (Ref. 15)
ALCL-5	ALCL	42.7	Crescenzo et al, 2015 (Ref. 15)
ALCL-6	ALCL	31.7	Crescenzo et al, 2015 (Ref. 15)
ALCL-7	ALCL	39.9	Crescenzo et al, 2015 (Ref. 15)
ALCL-8	ALCL	58.1	Crescenzo et al, 2015 (Ref. 15)
ALCL-9	ALCL	53.7	Crescenzo et al, 2015 (Ref. 15)
ALCL-10	ALCL	42.5	Crescenzo et al, 2015 (Ref. 15)
ALCL-11	ALCL	60.0	Crescenzo et al, 2015 (Ref. 15)
ALCL-12	ALCL	63.4	Crescenzo et al, 2015 (Ref. 15)
ALCL-13	ALCL	64.6	Crescenzo et al, 2015 (Ref. 15)
ALCL-14	ALCL	67.4	Crescenzo et al, 2015 (Ref. 15)
ALCL-15	ALCL	69.0	Crescenzo et al, 2015 (Ref. 15)
ALCL-16	ALCL	28.2	Crescenzo et al, 2015 (Ref. 15)
ALCL-17	ALCL	26.1	Crescenzo et al, 2015 (Ref. 15)
ALCL-18	ALCL	31.7	Crescenzo et al, 2015 (Ref. 15)
ALCL-19	ALCL	32.3	Crescenzo et al, 2015 (Ref. 15)
ALCL-20	ALCL	34.8	Crescenzo et al, 2015 (Ref. 15)
ALCL-21	ALCL	29.6	Crescenzo et al, 2015 (Ref. 15)
ALCL-22	ALCL	32.2	Crescenzo et al, 2015 (Ref. 15)
ALCL-23	ALCL	54.5	Crescenzo et al, 2015 (Ref. 15)
ALCL-24	ALCL	56.2	Palomero et al, 2014 (Ref. 14)
ALCL-25	ALCL	57.5	Palomero et al, 2014 (Ref. 14)
ALCL-26	ALCL	77.0	Palomero et al, 2014 (Ref. 14)
ALCL-27	ALCL	73.5	Palomero et al, 2014 (Ref. 14)
ALCL-28	ALCL	66.6	Palomero et al, 2014 (Ref. 14)
ALCL-29	ALCL	68.8	Palomero et al, 2014 (Ref. 14)
ALCL-30	ALCL	68.7	Palomero et al, 2014 (Ref. 14)
ALCL-31	ALCL	68.2	Palomero et al, 2014 (Ref. 14)
ALCL-32	ALCL	69.7	Palomero et al, 2014 (Ref. 14)
ALCL-33	ALCL	71.7	Palomero et al, 2014 (Ref. 14)
ALCL-34	ALCL	67.8	Palomero et al, 2014 (Ref. 14)
ALCL-35	ALCL	61.5	Palomero et al, 2014 (Ref. 14)
Fe_PD	ALCL cell line	91.3	New data
Karpas299	ALCL cell line	90.6	New data
L82	ALCL cell line	95.3	New data
MAC1	ALCL cell line	88.1	New data
SUDHL1	ALCL cell line	98.3	New data
TS	ALCL cell line	93.1	New data
T-cell-1	Naïve CD4+ T cells	118.3	Weinstein et al, 2014 (Ref. 16)
T-cell-2	Naïve CD4+ T cells	130.9	Weinstein et al, 2014 (Ref. 16)

T-cell-3	Naïve CD4+ T cells	65.2	Weinstein et al, 2014 (Ref. 16)
T-cell-4	Naïve CD4+ T cells	70.4	Weinstein et al, 2014 (Ref. 16)
T-cell-5	Effector T cells	104.2	Weinstein et al, 2014 (Ref. 16)
T-cell-6	Effector T cells	71.1	Weinstein et al, 2014 (Ref. 16)
T-cell-7	Effector T cells	74.1	Weinstein et al, 2014 (Ref. 16)
T-cell-8	Follicular helper T cells	111.1	Weinstein et al, 2014 (Ref. 16)
T-cell-9	Follicular helper T cells	118.8	Weinstein et al, 2014 (Ref. 16)
T-cell-10	Follicular helper T cells	84.5	Weinstein et al, 2014 (Ref. 16)
NKCL-1	NK cell lymphoma	97.9	Kucuk et al, 2015 (Ref. 12)
NKCL-2	NK cell lymphoma	96.5	Kucuk et al, 2015 (Ref. 12)
NKCL-3	NK cell lymphoma	104.1	Kucuk et al, 2015 (Ref. 12)
NKCL-4	NK cell lymphoma	88.3	Kucuk et al, 2015 (Ref. 12)
NKCL-5	NK cell lymphoma	97.7	Kucuk et al, 2015 (Ref. 12)
NKCL-6	NK cell lymphoma	105.6	Kucuk et al, 2015 (Ref. 12)
NKCL-7	NK cell lymphoma	97.3	Kucuk et al, 2015 (Ref. 12)
NKCL-8	NK cell lymphoma	115.8	Kucuk et al, 2015 (Ref. 12)
NKCL-10	NK cell lymphoma	94.0	Kucuk et al, 2015 (Ref. 12)
NKCL-11	NK cell lymphoma	102.5	Kucuk et al, 2015 (Ref. 12)
NKCL-12	NK cell lymphoma	98.2	Kucuk et al, 2015 (Ref. 12)
NKCL-13	NK cell lymphoma	104.1	Kucuk et al, 2015 (Ref. 12)
NKCL-14	NK cell lymphoma	97.8	Kucuk et al, 2015 (Ref. 12)
NKCL-16	NK cell lymphoma	124.8	Kucuk et al, 2015 (Ref. 12)
NKCL-17	NK cell lymphoma	112.6	Kucuk et al, 2015 (Ref. 12)
KHYG1	NK cell lymphoma cell line	79.1	Kucuk et al, 2015 (Ref. 12)
NKYS	NK cell lymphoma cell line	129.4	Kucuk et al, 2015 (Ref. 12)
PMIG-NK92	NK cell lymphoma cell line	96.9	Kucuk et al, 2015 (Ref. 12)
PRDM1-NK92	NK cell lymphoma cell line	99.7	Kucuk et al, 2015 (Ref. 12)
NK-cell-1	Normal NK cells	89.7	Weinstein et al, 2014 (Ref. 16)
NK-cell-2	Normal NK cells	124.2	Weinstein et al, 2014 (Ref. 16)
NK-cell-3	Normal NK cells	112.3	Weinstein et al, 2014 (Ref. 16)
PTCL-NOS-1	PTCL, NOS	66.0	Palomero et al, 2014 (Ref. 14)
PTCL-NOS-2	PTCL, NOS	66.5	Palomero et al, 2014 (Ref. 14)
PTCL-NOS-3	PTCL, NOS	64.6	Palomero et al, 2014 (Ref. 14)
PTCL-NOS-4	PTCL, NOS	65.2	Palomero et al, 2014 (Ref. 14)
PTCL-NOS-5	PTCL, NOS	60.5	Palomero et al, 2014 (Ref. 14)
PTCL-NOS-6	PTCL, NOS	62.9	Palomero et al, 2014 (Ref. 14)
PTCL-NOS-7	PTCL, NOS	66.7	Palomero et al, 2014 (Ref. 14)
PTCL-NOS-8	PTCL, NOS	83.4	Palomero et al, 2014 (Ref. 14)
PTCL-NOS-9	PTCL, NOS	67.0	Palomero et al, 2014 (Ref. 14)
PTCL-NOS-10	PTCL, NOS	68.5	Palomero et al, 2014 (Ref. 14)
PTCL-NOS-11	PTCL, NOS	116.4	New data
PTCL-NOS-12	PTCL, NOS	136.5	New data
PTCL-NOS-13	PTCL, NOS	151.7	New data

PTCL-NOS-14	PTCL, NOS	137.0	New data
PTCL-NOS-15	PTCL, NOS	120.2	New data
PTCL-NOS-16	PTCL, NOS	155.9	New data
PTCL-NOS-17	PTCL, NOS	125.5	New data

Table S2 Clonal assessment based on TCR CDR3 sequences and variable segment usage.

Case ID	Dominant TCRα%	TCRα CDR3 ratio	Dominant TCRβ%	TCRβ CDR3 ratio	Clonality based on CDR3	Vα ratio	Vβ ratio	Clonality based on V-usage	CDR3 & V-usage concordance
AITL-1	14%	4.0	17%	4.5	Monoclonal	4.0	2.3	Polyclonal	No
AITL-10	40%	10.7	58%	88.8	Monoclonal	63.2	28.7	Monoclonal	Yes
AITL-11	69%	115.7	80%	165.1	Monoclonal	41.9	24.1	Monoclonal	Yes
AITL-12	30%	10.2	74%	170.5	Monoclonal	2.9	44.8	Monoclonal	Yes
AITL-13	41%	12.9	44%	122.4	Monoclonal	21.8	12.5	Monoclonal	Yes
AITL-14	2%	2.1	4%	5.6	Polyclonal	3.0	3.1	Polyclonal	Yes
AITL-15	24%	10.9	1%	2.3	Monoclonal	15.3	2.5	Monoclonal	Yes
AITL-16	14%	7.9	29%	16.7	Monoclonal	7.6	5.9	Polyclonal	No
AITL-17	9%	4.5	5%	3.8	Polyclonal	3.3	3.2	Polyclonal	Yes
AITL-18	29%	8.9	73%	120.9	Monoclonal	30.7	54.5	Monoclonal	Yes
AITL-19	58%	46.0	37%	8.2	Monoclonal	19.2	8.9	Monoclonal	Yes
AITL-2	31%	14.2	42%	34.4	Monoclonal	14.2	19.7	Monoclonal	Yes
AITL-20	21%	12.7	47%	70.3	Monoclonal	6.2	14.1	Monoclonal	Yes
AITL-21	71%	19.8	95%	180.3	Monoclonal	31.1	41.9	Monoclonal	Yes
AITL-22	36%	24.1	21%	19.7	Monoclonal	10.1	9.2	Monoclonal	Yes
AITL-23	48%	23.2	80%	150.0	Monoclonal	7.1	5.5	Polyclonal	No
AITL-24	24%	18.4	81%	303.3	Monoclonal	6.9	43.2	Monoclonal	Yes
AITL-25	39%	17.0	53%	19.2	Monoclonal	9.2	15.2	Monoclonal	Yes
AITL-26	4%	3.0	10%	4.7	Polyclonal	2.2	7.6	Polyclonal	Yes
AITL-27	69%	27.9	90%	160.5	Monoclonal	19.2	93.9	Monoclonal	Yes
AITL-28	79%	11.5	92%	90.0	Monoclonal	18.7	48.0	Monoclonal	Yes
AITL-29	67%	17.2	81%	74.5	Monoclonal	18.3	47.5	Monoclonal	Yes
AITL-3	15%	18.6	3%	2.7	Monoclonal	4.0	3.8	Polyclonal	No
AITL-30	3%	5.0	1%	2.0	Polyclonal	3.5	6.5	Polyclonal	Yes
AITL-31	10%	4.2	42%	14.6	Monoclonal	2.2	11.9	Monoclonal	Yes
AITL-32	46%	61.8	4%	5.3	Monoclonal	13.5	30.1	Monoclonal	Yes
AITL-33	5%	4.5	34%	37.1	Monoclonal	2.8	11.0	Monoclonal	Yes
AITL-34	47%	24.7	34%	7.0	Monoclonal	6.3	6.7	Polyclonal	No
AITL-35	35%	5.0	62%	36.7	Monoclonal	16.5	21.4	Monoclonal	Yes
AITL-36	8%	4.7	16%	7.6	Monoclonal	2.4	5.9	Polyclonal	No
AITL-37	60%	29.9	12%	3.4	Monoclonal	15.4	5.7	Monoclonal	Yes
AITL-38	43%	63.9	61%	44.8	Monoclonal	23.4	23.7	Monoclonal	Yes
AITL-39	77%	103.1	78%	124.7	Monoclonal	52.8	21.9	Monoclonal	Yes
AITL-4	11%	2.8	9%	2.2	Monoclonal	3.1	3.9	Polyclonal	No
AITL-40	55%	34.1	66%	71.9	Monoclonal	12.8	65.0	Monoclonal	Yes
AITL-5	38%	17.6	80%	97.8	Monoclonal	5.3	44.9	Monoclonal	Yes
AITL-6	74%	26.8	7%	2.6	Monoclonal	23.2	6.6	Monoclonal	Yes
AITL-7	11%	14.3	5%	6.9	Monoclonal	6.0	5.7	Polyclonal	No
AITL-8	33%	18.9	9%	2.4	Monoclonal	2.6	5.1	Polyclonal	No
AITL-9	90%	65.1	22%	10.3	Monoclonal	10.7	18.7	Monoclonal	Yes
ALCL-1	98%	447.5	99%	364.0	Monoclonal	42.4	70.0	Monoclonal	Yes
ALCL-12	1%	3.3	1%	2.6	Polyclonal	3.8	4.8	Polyclonal	Yes
ALCL-16	60%	24.9	81%	105.7	Monoclonal	40.2	186.2	Monoclonal	Yes
ALCL-17	67%	74.5	80%	52.3	Monoclonal	27.3	42.5	Monoclonal	Yes
ALCL-18	73%	54.0	62%	51.0	Monoclonal	20.3	47.5	Monoclonal	Yes
ALCL-19	2%	3.5	1%	3.0	Polyclonal	2.5	3.6	Polyclonal	Yes
ALCL-20	37%	9.9	18%	10.5	Monoclonal	2.0	3.9	Polyclonal	No
ALCL-21	52%	83.7	73%	147.8	Monoclonal	2.4	23.2	Monoclonal	Yes
ALCL-23	24%	9.1	56%	80.5	Monoclonal	3.0	31.4	Monoclonal	Yes
ALCL-24	94%	153.0	94%	23.4	Monoclonal	55.5	16.5	Monoclonal	Yes
ALCL-25	34%	43.3	3%	4.5	Monoclonal	65.6	2.8	Monoclonal	Yes
ALCL-26	4%	3.0	69%	62.0	Monoclonal	2.3	36.5	Monoclonal	Yes
ALCL-27	56%	11.5	62%	30.2	Monoclonal	22.1	12.0	Monoclonal	Yes
ALCL-28	4%	3.4	11%	5.3	Monoclonal	3.0	3.5	Polyclonal	No
ALCL-29	69%	14.7	92%	643.6	Monoclonal	174.7	131.5	Monoclonal	Yes
ALCL-32	1%	2.6	1%	2.2	Polyclonal	3.1	2.6	Polyclonal	Yes
ALCL-34	3%	3.4	1%	2.4	Polyclonal	2.4	2.5	Polyclonal	Yes

ALCL-35	91%	190.8	7%	2.8	Monoclonal	10.3	2.4	Monoclonal	Yes
ALCL-5	84%	92.4	83%	60.3	Monoclonal	78.4	89.9	Monoclonal	Yes
ALCL-6	10%	5.3	11%	3.8	Monoclonal	3.3	6.1	Polyclonal	No
ALCL-8	85%	54.4	70%	17.6	Monoclonal	12.6	6.2	Monoclonal	Yes
ALCL-9	66%	3.4	51%	3.0	Monoclonal	2.8	4.0	Polyclonal	No
L82	88%	15.0	100%	1499.0	Monoclonal	2.5	2057.7	Monoclonal	Yes
PTCL-NOS-1	53%	19.7	52%	59.8	Monoclonal	12.7	8.4	Monoclonal	Yes
PTCL-NOS-10	50%	155.3	60%	97.7	Monoclonal	6.2	4.6	Polyclonal	No
PTCL-NOS-11	95%	361.4	89%	144.2	Monoclonal	7.5	319.9	Monoclonal	Yes
PTCL-NOS-13	95%	578.5	96%	167.1	Monoclonal	262.5	8.8	Monoclonal	Yes
PTCL-NOS-14	3%	2.3	5%	2.5	Polyclonal	3.9	6.6	Polyclonal	Yes
PTCL-NOS-15	42%	5.8	46%	7.4	Monoclonal	2.5	21.8	Monoclonal	Yes
PTCL-NOS-16	42%	5.4	74%	34.7	Monoclonal	7.6	26.4	Monoclonal	Yes
PTCL-NOS-17	3%	2.4	2%	2.9	Polyclonal	3.4	9.5	Polyclonal	Yes
PTCL-NOS-2	14%	22.6	3%	3.7	Monoclonal	12.3	3.8	Monoclonal	Yes
PTCL-NOS-3	18%	24.4	18%	6.5	Monoclonal	9.4	5.3	Polyclonal	No
PTCL-NOS-4	14%	25.6	8%	4.5	Monoclonal	8.0	4.4	Polyclonal	No
PTCL-NOS-5	7%	3.9	8%	4.5	Polyclonal	3.7	3.7	Polyclonal	Yes
PTCL-NOS-7	20%	42.5	20%	26.2	Monoclonal	7.0	13.9	Monoclonal	Yes
PTCL-NOS-9	77%	23.7	65%	8.1	Monoclonal	2.7	20.1	Monoclonal	Yes
T-cell-1	0%	2.0	0%	2.3	Normal T cells	2.6	3.8	Normal T cells	Yes
T-cell-10	2%	2.9	2%	2.4	Normal T cells	2.5	3.9	Normal T cells	Yes
T-cell-2	0%	2.0	0%	2.0	Normal T cells	2.1	3.7	Normal T cells	Yes
T-cell-3	0%	2.0	0%	2.0	Normal T cells	2.3	3.1	Normal T cells	Yes
T-cell-4	1%	2.3	0%	2.4	Normal T cells	2.1	4.7	Normal T cells	Yes
T-cell-5	0%	2.8	0%	3.0	Normal T cells	2.5	2.8	Normal T cells	Yes
T-cell-6	1%	2.2	1%	2.4	Normal T cells	2.5	5.2	Normal T cells	Yes
T-cell-7	0%	2.3	0%	2.4	Normal T cells	2.7	3.0	Normal T cells	Yes
T-cell-8	1%	2.5	1%	2.7	Normal T cells	2.2	4.5	Normal T cells	Yes
T-cell-9	1%	2.4	1%	2.6	Normal T cells	2.2	3.5	Normal T cells	Yes

Table S3 List of samples with biallelic TCR strands.

Original ID	Chain	C1%	C2%	C3%	C1/C2 Ratio	C2/C3 Ratio	Out-of-frame allele
AITL-2	TCRα	15.62%	15.11%	2.17%	1.0	7.0	No
AITL-11	TCRα	51.98%	16.60%	0.59%	3.1	28.0	Yes
AITL-19	TCRα	47.04%	11.19%	1.27%	4.2	8.8	Yes
AITL-22	TCRβ	14.67%	6.40%	1.07%	2.3	6.0	No
AITL-23	TCRβ	70.74%	9.04%	0.53%	7.8	17.0	Yes
AITL-24	TCRα	12.43%	11.50%	1.30%	1.1	8.9	Yes
AITL-27	TCRα	54.55%	14.60%	2.48%	3.7	5.9	Yes
AITL-32	TCRα	31.30%	14.44%	0.74%	2.2	19.5	Yes
AITL-34	TCRα	24.63%	22.11%	1.89%	1.1	11.7	No
AITL-37	TCRα	38.65%	21.80%	2.02%	1.8	10.8	No
AITL-39	TCRα	71.88%	5.12%	0.75%	14.0	6.9	Yes
AITL-39	TCRβ	73.96%	3.67%	0.62%	20.2	5.9	Yes
ALCL-8	TCRα	63.81%	20.82%	1.56%	3.1	13.4	No
ALCL-16	TCRα	41.28%	18.56%	2.40%	2.2	7.7	Yes
ALCL-18	TCRα	63.51%	9.46%	1.35%	6.7	7.0	Yes
ALCL-24	TCRα	87.96%	5.71%	0.61%	15.4	9.3	Yes
ALCL-27	TCRβ	40.33%	21.25%	2.04%	1.9	10.4	No
ALCL-35	TCRα	88.20%	3.11%	0.48%	28.4	6.5	Yes
PTCL-NOS-1	TCRα	30.60%	22.57%	2.70%	1.4	8.3	No
PTCL-NOS-1	TCRβ	28.09%	23.70%	0.87%	1.2	27.4	No
PTCL-NOS-7	TCRα	16.84%	3.26%	0.47%	5.2	6.9	Yes
PTCL-NOS-11	TCRα	91.22%	3.40%	0.26%	26.8	13.0	Yes
PTCL-NOS-13	TCRα	90.57%	4.09%	0.16%	22.1	25.0	Yes
PTCL-NOS-13	TCRβ	82.60%	13.00%	0.57%	6.4	22.7	Yes
